# Research Trends and Hotspots in Female Pattern Hair Loss: A Bibliometric Study

**DOI:** 10.1111/jocd.70369

**Published:** 2025-07-30

**Authors:** Hongjuan Fu

**Affiliations:** ^1^ Jinhua Fifth Hospital Jinhua China

**Keywords:** alopecia, bibliometric analysis, CiteSpace, female pattern hair loss

## Abstract

**Background:**

Female pattern hair loss (FPHL) is a common yet understudied condition with significant psychosocial impacts. Understanding global research trends and emerging topics in FPHL is essential for guiding future investigations.

**Aims:**

This bibliometric analysis aimed to provide an overview of key trends, influential contributors, and evolving research themes in FPHL from 1957 to 2024.

**Patients/Methods:**

Publications related to FPHL were retrieved from the Web of Science Core Collection. Bibliometric analysis and visualization were performed using VOSviewer, CiteSpace, and the R package “bibliometrix.”

**Results:**

The study analyzed 488 publications authored by 2165 researchers across 189 journals. The USA led in publication output and citations, followed by China and South Korea. Leading institutions included the Egyptian Knowledge Bank, University of Melbourne, and National Taiwan University. Shapiro Jerry was the most prolific author. The Journal of Cosmetic Dermatology had the highest publication count, whereas the Journal of the American Academy of Dermatology was the most cited. Keyword analysis identified emerging hotspots, such as “mechanisms,” “management,” and “safety,” with growing interest in “scalp,” “association,” and “telogen effluvium.”

**Conclusions:**

This study highlights the global research landscape of FPHL, emphasizing the focus on treatment efficacy, safety, and underlying mechanisms. Future research may prioritize accurate diagnostic methods and risk factor exploration.

## 1 Introduction

Female pattern hair loss (FPHL) is a prevalent form of hair loss among women, characterized primarily by a gradual thinning of hair at the crown, whereas the frontal hairline typically remains intact [1]. The manifestations of FPHL can vary considerably among individuals but generally include reduced hair density, thinning of hair shafts, and increased scalp visibility [2]. The condition is particularly common in women over the age of 50, with a significantly higher incidence observed in postmenopausal women [3, 4]. The etiology of FPHL is multifactorial, with genetic predisposition and androgenic influences being the most prominent factors. Genetic susceptibility can increase an individual's sensitivity to androgens, which in turn leads to the progressive miniaturization of hair follicles, along with the thinning, shortening, and eventual loss of hair [5, 6]. Hormonal imbalances, particularly elevated androgen levels, are also implicated in FPHL, as seen in conditions, such as polycystic ovary syndrome, where these levels often play a contributing role [7].

Treatment options for FPHL are diverse, ranging from topical therapies to advanced medical procedures [8, 9]. The only topical agent currently approved by the USA Food and Drug Administration for the treatment of FPHL is minoxidil, which has demonstrated efficacy, particularly at a concentration of 2% [10]. Low‐level laser therapy (LLLT) has been found to enhance the effects of minoxidil when used in combination [11], whereas botulinum toxin A offers an alternative for patients who experience allergic reactions to minoxidil [12]. Platelet‐rich plasma therapy, which involves the injection of growth factor‐enriched platelets to stimulate hair follicle regeneration, has shown promising results in some cases, surpassing the effectiveness of minoxidil [13]. Oral medications such as finasteride and spironolactone have proven beneficial for select patients, with spironolactone showing superior results [14]. Additional interventions, such as microneedling combined with LLLT have been found to significantly increase hair density, whereas scalp micropigmentation offers a nonsurgical alternative for patients seeking a cosmetic solution to hair loss [15]. The treatment approach for FPHL should be individualized, as the condition's progression and the response to treatment can vary. Nonetheless, further research is necessary to establish the long‐term efficacy and safety of these therapeutic interventions and to refine personalized treatment strategies. Bibliometric analysis provides a quantitative approach to assess the evolution and emerging trends within a research field. Although there are several bibliometric studies focusing on related conditions, such as androgenetic alopecia [16, 17, 18] and alopecia areata [19, 20, 21], a comprehensive bibliometric analysis dedicated exclusively to FPHL is notably absent in the literature. This gap in the existing literature highlights the necessity of conducting a bibliometric analysis of FPHL. The aim of this study is to conduct a comprehensive bibliometric analysis of research trends concerning FPHL in order to identify key research trends, influential publications, and future directions.

## 2 Materials and Methods

### 2.1 Literature Search

A comprehensive literature search was conducted using the Web of Science Core Collection (WoSCC) database, focusing on studies related to FPHL from January 1, 1957, to October 12, 2024. The search strategy utilized the following search terms: TS = (Female pattern hair loss OR Female‐pattern hair loss OR Female Pattern Baldness OR Baldness, Female Pattern OR FPHL) [22]. The literature search was restricted to English‐language publications to maintain linguistic consistency, and only articles were included to ensure a uniform publication type. Data extraction was carried out in “Full record and cited references” and “plain text” formats to capture comprehensive information, including the number of publications, citations, titles, author details, institutional affiliations, countries/regions, keywords, and journals.

### 2.2 Statistical Analysis

The bibliometric analysis was conducted using a suite of analytical and visualization tools to ensure robust data insights. VOSviewer (Version 1.6.20), CiteSpace (Version 6.3.R1), and the R package “bibliometrix” (R 4.3.3) were employed to facilitate a multidimensional analysis of the dataset.

VOSviewer was primarily used for mapping collaboration networks, including coauthorship, citation, and co‐citation analyses. The size of nodes in VOSviewer represents the frequency of occurrence, whereas the links between nodes indicate the strength of collaboration or co‐occurrence. Keyword co‐occurrence analysis was also performed in VOSviewer to reveal research hotspots and trends within FPHL studies. CiteSpace was used to analyze temporal trends and emerging research frontiers through keyword burst detection. The time period was set from January 1994 to October 2024, with keywords having a threshold of five. The network was pruned using the Pathfinder and Pruning Merge algorithms. The final output was a keyword burst timeline, visually depicting the evolution of key topics in the field of FPHL. The R package “bibliometrix” was employed for trend mapping and ranking analyses, allowing for the tracking of publication and citation patterns across authors, institutions, and countries. In addition, various bibliometric indices, including the H‐index for evaluation of productivity and citation impact [23, 24], along with the Impact Factor (IF) of journals obtained from the latest Journal Citation Reports (JCR), were used to assess journal and author significance.

## 3 Results

### 3.1 The Publication Trends

**FIGURE 1:**
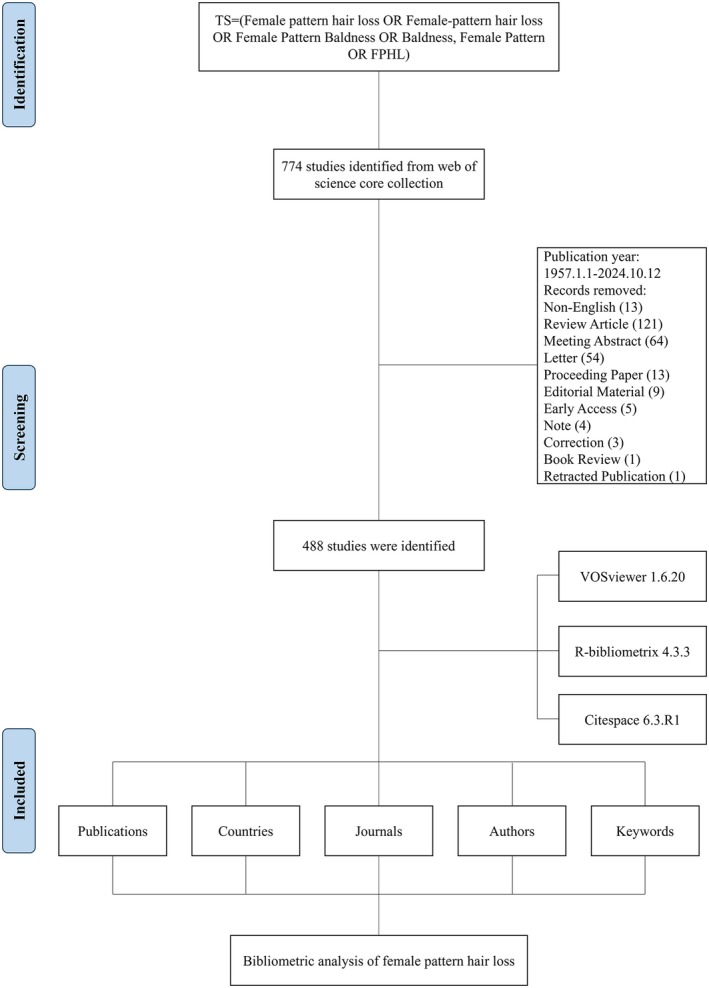
Flowchart of data screening process.

**FIGURE 2:**
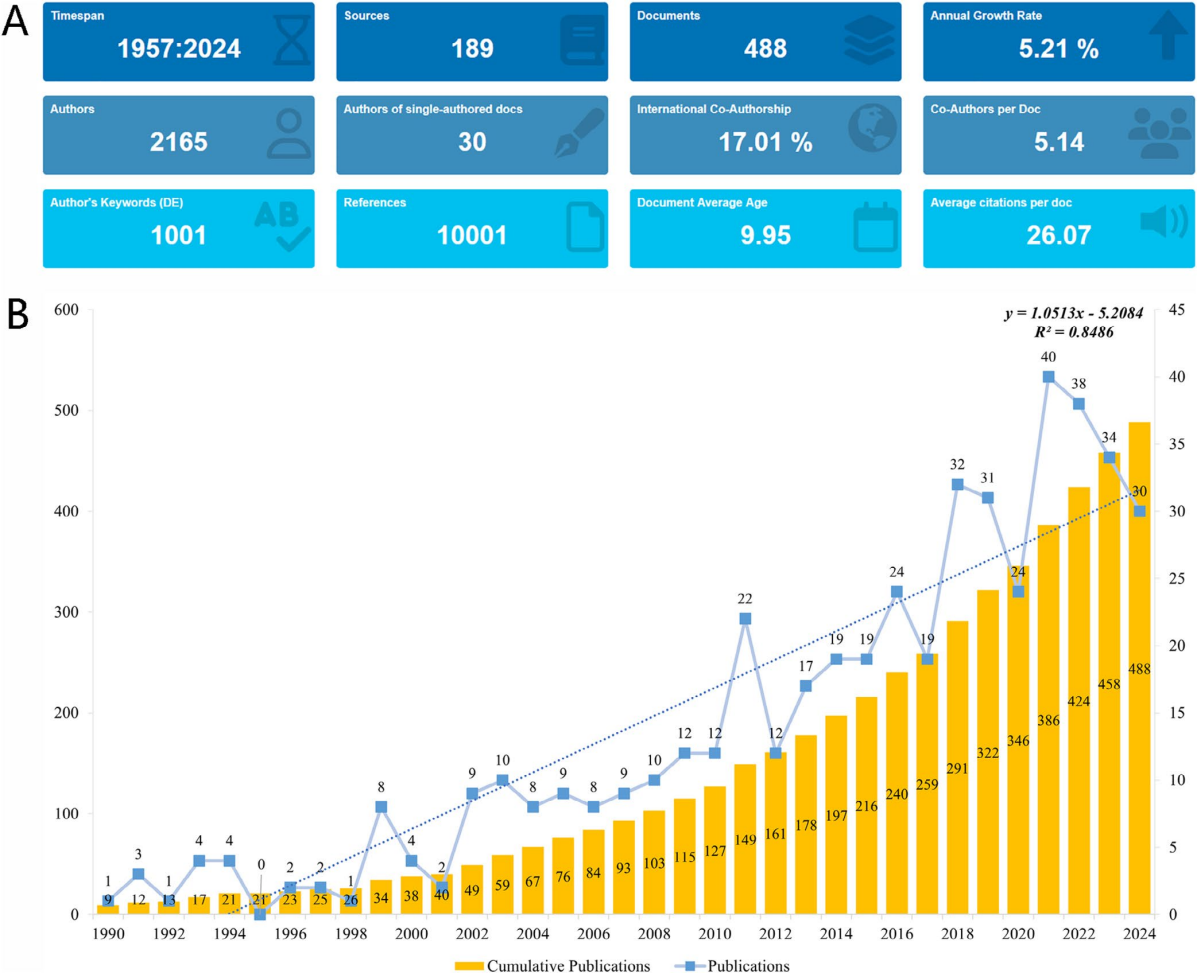
The characteristics of the document in FPHL. (A) Comprehensive overview of the bibliometric analysis. (B) Annual growth of publications on FPHL.

A detailed flowchart illustrating the literature screening process was presented in Figure 1. A total of 488 publications from 1957 to 2024 were included in the analysis, contributed by 2165 authors from 189 journals across 202 countries/regions. These works were published in 1336 institutions, citing 10 001 references. The number of publications exhibited a general upward trend with an annual growth rate of 5.21% (Figure 2A). The trend in publication numbers was illustrated in Figure 2B. Specifically, from 1990 to 2001, the number of publications remained relatively stable, fluctuating between 0 and 8. However, after 2002, there was a significant increase, peaking in 2021 with 40 publications (Figure 2B).

### 3.2 Analysis of Leading Countries

**FIGURE 3:**
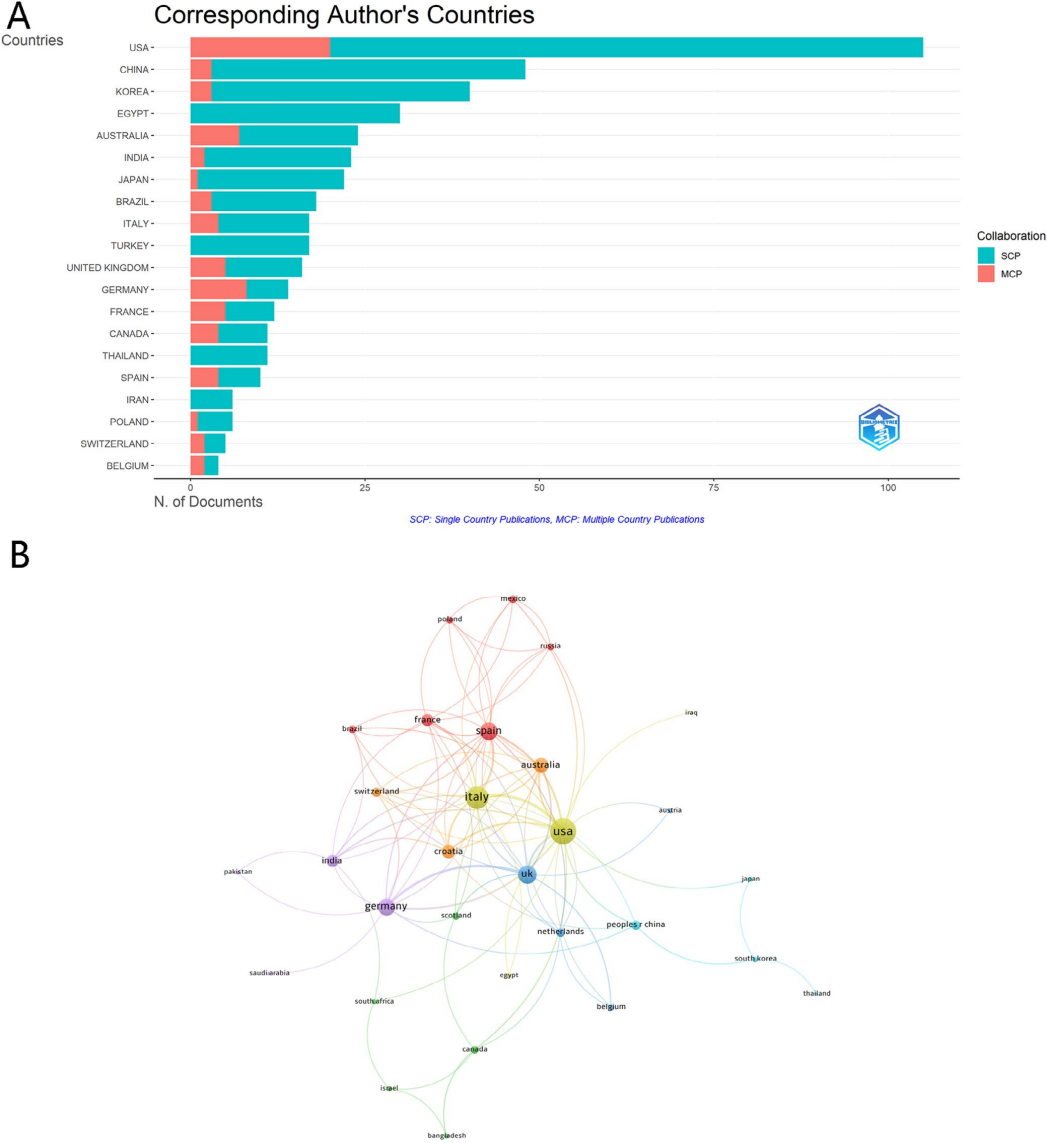
Global distribution and collaboration in FPHL. (A) Distribution of Corresponding Authors' Publications by Country. (B) Visualization Map Depicting the Collaboration Among Different Countries.

The global distribution of publications in this field spanned multiple countries, with notable contributions from the USA, China, and South Korea. As shown in Table 1, the USA led with 105 articles, accounting for 21.5% of the total, followed by China (48, 9.8%) and South Korea (40, 8.2%). The USA recorded the highest total publications and total citations, with 317 and 3936, respectively. The analysis also highlighted international collaborations. The USA had the most multiple‐country publications (*n* = 20), indicating a substantial level of international cooperation, followed by Germany (*n* = 8) and Australia (*n* = 7) (Figure 3A). Among the 33 countries involved in international collaborations, with at least two articles, the USA had the highest number of collaborations with other countries (link strength = 70), followed by Italy (link strength = 52) and the United Kingdom (link strength = 34) (Figure 3B).

**TABLE 1:** Publication and citation profiles of leading countries.

Country	Articles	Freq	SCP	MCP	MCP_ratio	TP	TP_rank	TC	TC_rank	Average citations
USA	105	21.5	85	20	0.190	317	1	3936	1	37.5
China	48	9.8	45	3	0.063	144	2	531	6	11.1
South Korea	40	8.2	37	3	0.075	129	3	965	3	24.1
Egypt	30	6.1	30	0	0.000	67	6	424	8	14.1
Australia	24	4.9	17	7	0.292	87	4	998	2	41.6
India	23	4.7	21	2	0.087	67	8	201	14	8.7
Japan	22	4.5	21	1	0.045	79	5	363	9	16.5
Brazil	18	3.7	15	3	0.167	45	11	268	12	14.9
Italy	17	3.5	13	4	0.235	49	12	560	5	32.9
Turkey	17	3.5	17	0	0.000	40	14	190	16	11.2
United Kingdom	16	3.3	11	5	0.313	59	9	693	4	43.3
Germany	14	2.9	6	8	0.571	67	7	293	10	20.9
France	12	2.5	7	5	0.417	41	13	491	7	40.9
Canada	11	2.3	7	4	0.364	30	15	288	11	26.2
Thailand	11	2.3	11	0	0.000	20	17	184	18	16.7
Spain	10	2	6	4	0.400	45	12	242	13	24.2
Iran	6	1.2	6	0	0.000	19	18	44	22	7.3
Poland	6	1.2	5	1	0.167	23	16	17	29	2.8
Switzerland	5	1	3	2	0.400	6	24	175	19	35
Belgium	4	0.8	2	2	0.500	10	20	193	15	48.2

*Note:* Articles: Publications of corresponding authors only.

Abbreviations: Average Citations, The average number of citations per publication; Freq, frequency of total publications; MCP, multiple country publications; MCP_Ratio, proportion of multiple country publications; SCP, single country publications; TC, total citations; TC_rank, rank of total citations; TP, total publications; TP_rank, rank of total publications.

### 3.3 Analysis of Leading Institutions

**FIGURE 4:**
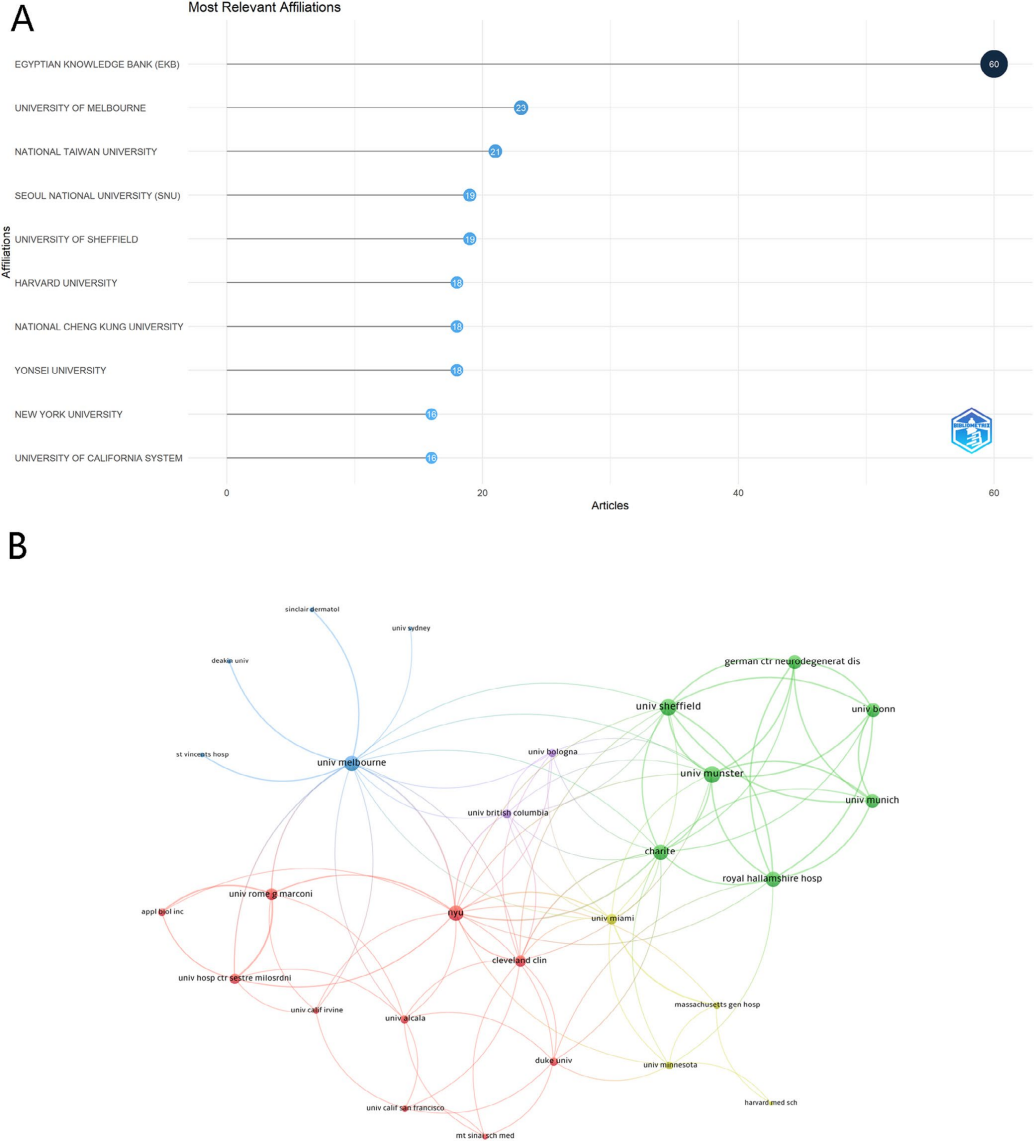
Institutional contributions and collaborations in FPHL. (A) Top 10 Institutions by Article Count and Rank. (B) Visualization Map Depicting the Collaboration Among Different Institutions.

A total of 189 institutions conducted research on FPHL. To explore the contributions of these institutions, the number of publications from various institutions was analyzed. The top 10 most productive institutions are displayed in Figure 4A. Among them, three research institutions were from the USA, two from China, two from South Korea, and one each from Egypt, Australia, and the United Kingdom. The Egyptian Knowledge Bank stood out with 60 publications, followed by the University of Melbourne (*n* = 23) and National Taiwan University (*n* = 21) (Figure 4A).

Among the 71 institutions involved in international collaborations with at least three articles, University of Münster and University of Sheffield had the highest number of collaborations with other institutions (link strength = 30), followed by New York University (link strength = 27). This reflected a broad engagement across multiple countries, enhancing the exchange of knowledge and expertise. The collaboration network also highlighted the central role of Europe and the USA (Figure 4B).

### 3.4 Analysis of Authors and Co‐Cited Authors

A total of 2165 authors have contributed to the development of research in this field. As summarized in Table 2, Shapiro Jerry ranked highest in total publications (*n* = 9), followed by Suchonwanit Poonkiat (*n* = 8) and Tsuboi Ryoji (*n* = 7). In terms of total citations, Suchonwanit Poonkiat led with 444 total citations, followed by Messenger AG (*n* = 321) and Shapiro Jerry (*n* = 262). In addition, Shapiro Jerry also had the highest h‐index with seven, followed by Tosti Antonella with an h‐index of six.

**TABLE 2:** Publication and citation profiles of high‐impact authors.

Authors	h_index	g‐index	m‐index	PY_start	TP	TP_Frac	TP_rank	TC	TC_rank
Shapiro Jerry	7	9	0.58	2013	9	1.46	1	262	3
Tosti Antonella	6	6	0.60	2015	6	1.23	5	121	13
Blume‐Peytavi Ulrike	5	5	0.36	2011	5	0.57	6	135	11
Sinclair R	5	5	0.23	2003	5	2.06	10	444	1
Suchonwanit Poonkiat	5	8	0.71	2018	8	2.13	2	161	8
Tsuboi Ryoji	5	7	0.28	2007	7	0.88	3	138	10
Goren Andy	4	5	0.29	2011	5	1.03	7	36	23
Hu Ruiming	4	4	0.40	2015	4	0.45	14	26	25
Kim Seong‐Jin	4	4	0.22	2007	4	0.72	16	183	7
Lee Won‐Soo	4	5	0.22	2007	5	0.76	8	152	9
Lutz Gerhard	4	4	0.31	2012	4	0.27	20	54	15
Messenger AG	4	4	0.15	1999	4	1.25	22	321	2
Sheng Youyu	4	4	0.40	2015	4	0.45	25	26	25
Sinclair Rodney	4	5	0.22	2007	5	1.40	11	94	14
Tosti A	4	4	0.15	1999	4	1.14	27	188	6
Yang Qinping	4	4	0.40	2015	4	0.45	29	26	25
Aktan Sebnem	3	3	0.17	2007	3	0.78	30	52	16
Becker Tim	3	3	0.23	2012	3	0.16	31	39	17
Bergfeld Wilma	3	4	0.27	2014	4	0.74	12	190	5
Betz Regina C.	3	3	0.23	2012	3	0.16	32	39	17

Abbreviations: g_index, the g‐index of the journal, which gives more weight to highly cited articles; H_index, the h‐index of the journal, which measures both the productivity and citation impact of the publications; m_index, the m‐index of the journal, which is the h‐index divided by the number of years since the first published paper; PY_start, publication year start, indicating the year the journal started publication; TC, total citations; TC_rank, rank of total citations; TP, total publications; TP_Frac, fraction of total publications; TP_rank, rank of total publications.

**FIGURE 5:**
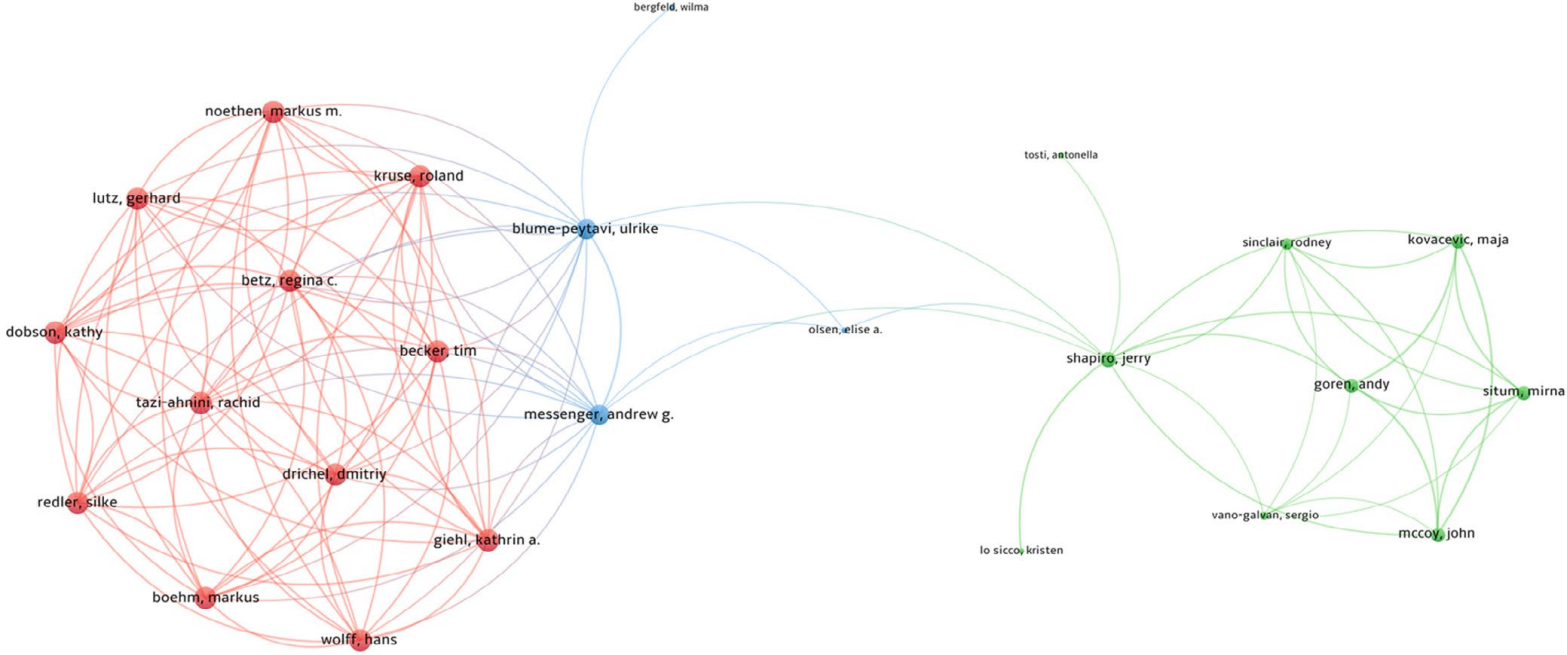
Author collaboration network in FPHL.

The collaborative relationships among researchers were illustrated with 69 authors who have a minimum of three articles, as shown in Figure 5. Specifically, we observed close collaboration among multiple authors: Becker Tim, Betz Regina C., Boehm Markus, Dobson Kathy, Drichel Dmitriy, Giehl Kathrin A., Kruse Roland, Lutz Gerhard, Noethen Markus M., Redler Silke, and Tazi‐ahnini Rachid had the highest number of collaborations with other authors (all link strengths = 37) (Figure 5).

### 3.5 Analysis of Journals and Co‐Cited Journals

The research publications in this domain were distributed across 1336 journals, with the *Journal of Cosmetic Dermatology* leading with 37 publications, followed by the *Journal of the American Academy of Dermatology* (*n* = 29) and the *British Journal of Dermatology* (*n* = 22) (Table 3). Two of these journals are classified in JCR Q1, whereas one is categorized in JCR Q2. In terms of total citations, the *Journal of the American Academy of Dermatology* stood out with 1232 citations, followed by the *British Journal of Dermatology* (*n* = 963). The *Journal of the American Academy of Dermatology* also held the highest IF at 12.8 among the top 20 impactful authors, underscoring its significant influence. Other journals, such as the *British Journal of Dermatology* (IF = 11.0) and the *Journal of the European Academy of Dermatology and Venereology* (IF = 8.4), also maintained high IF values, reinforcing their relevance and authority in dermatological research (Table 3).

**TABLE 3:** Bibliometric indicators of high‐impact journals.

Journal	h_index	g‐index	m‐index	TP	TP_rank	TC	TC_rank	PY_start	IF_2023	JCR_2023
*Journal of the American Academy of Dermatology*	21	29	0.656	29	2	1232	1	1993	12.8	1
*British Journal of Dermatology*	19	22	0.576	22	3	963	2	1992	11.0	1
*International Journal of Dermatology*	11	22	0.423	22	4	214	7	1999	3.5	1
*Journal of Cosmetic Dermatology*	10	16	0.769	37	1	118	15	2012	2.3	2
*Journal of the European Academy of Dermatology and Venereology*	9	11	0.346	11	8	214	8	1999	8.4	1
*Journal of Dermatology*	8	12	0.444	12	7	98	20	2007	2.9	2
*Archives of Dermatological Research*	7	13	0.194	13	5	74	25	1989	1.8	3
*Dermatologic Therapy*	7	12	0.412	13	6	142	11	2008	3.7	1
*Dermatology*	7	10	0.241	10	9	131	12	1996	3.0	2
*Journal of Clinical Endocrinology & Metabolism*	6	6	0.091	6	17	220	6	1959	5.0	1
*Journal of Drugs in Dermatology*	6	8	0.429	8	14	68	30	2011	1.5	3
*Annals of Dermatology*	5	8	0.385	8	11	60	38	2012	1.5	3
*Australasian Journal of Dermatology*	5	8	0.278	8	12	84	22	2007	2.2	2
*Dermatologic Surgery*	5	9	0.200	9	10	339	4	2000	2.5	1
*Indian Journal of Dermatology Venereology & Leprology*	5	6	0.294	6	16	64	33	2008	3.2	2
*Journal of Dermatological Science*	5	6	0.227	6	18	79	24	2003	3.8	1
*Anais Brasileiros de Dermatologia*	4	4	0.308	4	25	70	29	2012	2.6	2
*Clinical Cosmetic and Investigational Dermatology*	4	7	0.571	8	13	32	64	2018	1.9	3
*Dermatologic Clinics*	4	4	0.200	4	26	106	18	2005	2.2	2
*Experimental Dermatology*	4	5	0.174	5	20	129	13	2002	3.5	1

Abbreviations: g_index, the g‐index of the journal, which gives more weight to highly‐cited articles; h_index, the h‐index of the journal, which measures both the productivity and citation impact of the publications; IF_2023, Impact Factor in 2023, indicating the average number of citations to recent articles published in the journal; JCR_2023, the quartile ranking of the journal in the Journal Citation Reports in 2023, indicating the journal's ranking relative to others in the same field (Q1, top 25%, Q2, 25%–50%, Q3, 50%–75%, Q4, bottom 25%); m_index, the m‐index of the journal, which is the h‐index divided by the number of years since the first published paper; PY_start, publication year start, indicating the year the journal started publication; TC, total citations; TC_rank, rank of total citations; TP, total publications; TP_rank, rank of total publications.

**FIGURE 6:**
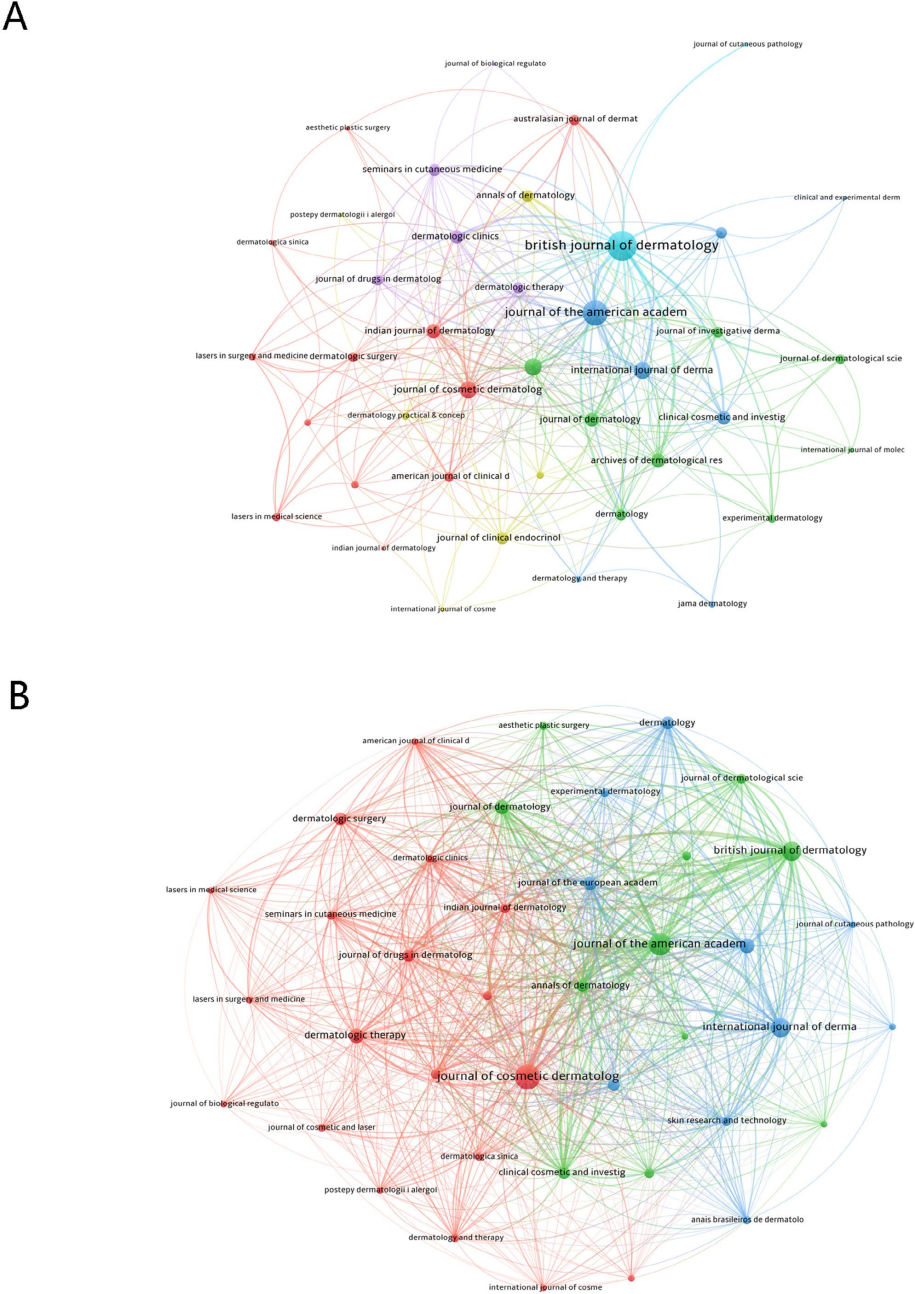
Network analyses of journals in FPHL. (A) The Co‐occurrence Networks of Journals. (B) The Coupling Networks of Journals.

The co‐occurrence networks of journals included 41 journals with at least three occurrences. The three key journals with the highest total link strength in the co‐occurrence networks were the *British Journal of Dermatology* (1553), the *Journal of the American Academy of Dermatology* (1420), and the *International Journal of Dermatology* (603) (Figure 6A). Similarly, the coupling networks of journals also included 41 journals with at least three couplings. The three key journals with the highest total link strength in this analysis were the *Journal of the American Academy of Dermatology* (link strength = 61 358), the *British Journal of Dermatology* (link strength = 43 219), and the *Journal of Cosmetic Dermatology* (link strength = 40 006) (Figure 6B).

### 3.6 Analysis of Co‐Occurring Keywords and Burst Terms

**FIGURE 7:**
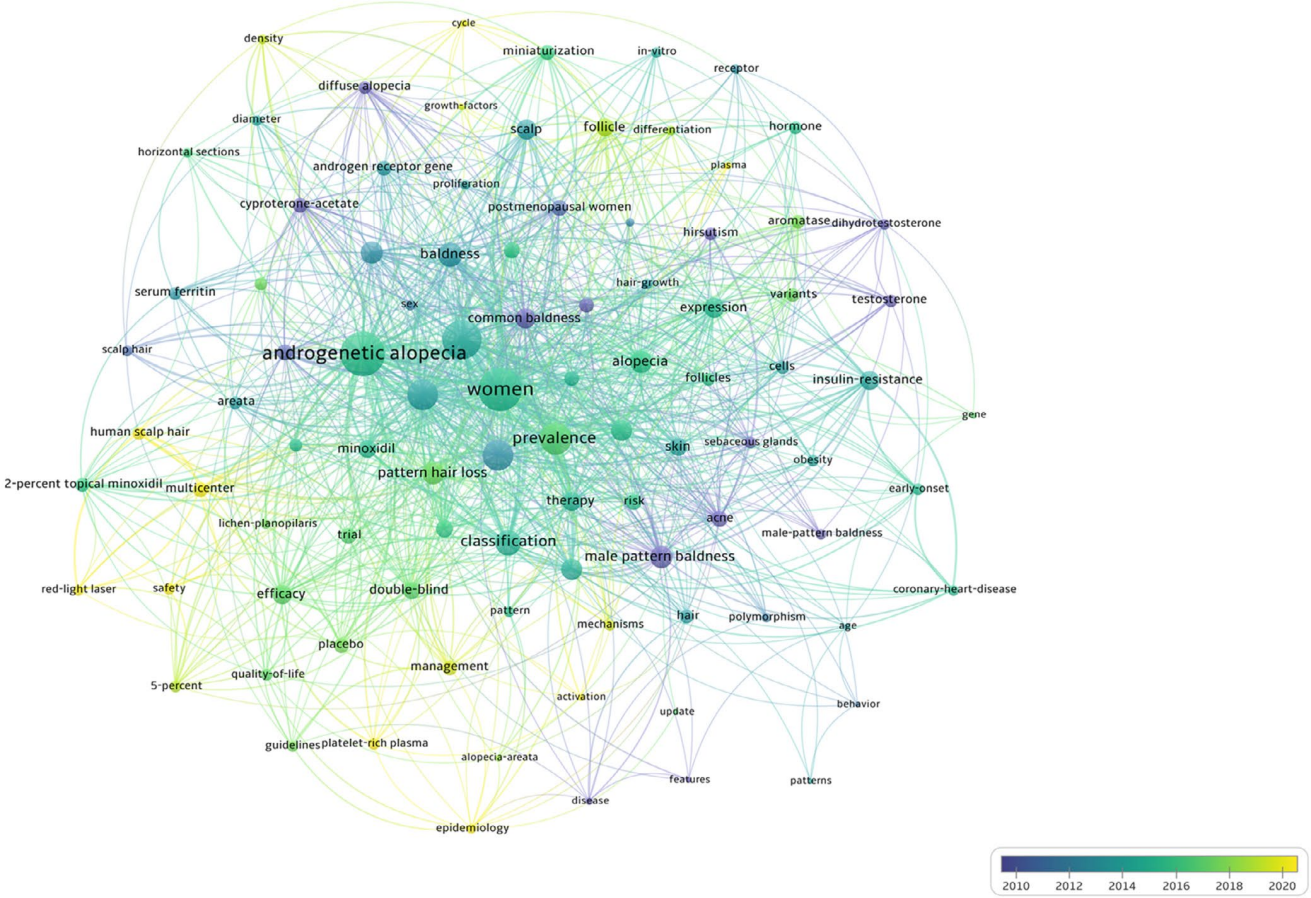
Keyword co‐occurrence network in FPHL.

The keyword analysis offers insights into research hotspots and thematic trends within the field. The keyword co‐occurrence network, visualized in Figure 7, illustrates the relationships between key topics, with node size representing frequency and color indicating the average year of publication. Early research, indicated by darker nodes, focused on broad themes such as “common baldness” (18 occurrences, link strength = 88) and “prevalence” (51 occurrences, link strength = 216). In contrast, recent studies, represented by yellow nodes, have shifted toward more specific topics, such as “mechanisms,” “management,” and “safety,” suggesting an increasing specialization in therapeutic factors related to FPHL (Table 4 and Figure 7).

**TABLE 4:** Top 20 keywords in FPHL research.

Id	Keyword	Occurrences	Total link strength
68	Androgenetic alopecia	123	423
1063	Women	98	393
614	Men	69	319
788	Prevalence	51	216
392	Finasteride	39	200
437	Growth	48	195
198	Classification	36	140
113	Baldness	34	124
591	Male pattern baldness	32	108
728	Pattern hair loss	36	108
998	Topical minoxidil	20	107
58	Alopecia	35	103
455	Hair loss	27	96
105	Association	21	92
214	Common baldness	18	88
364	Expression	24	88
866	Scalp	22	83
323	Efficacy	19	81
985	Therapy	16	76
539	Insulin‐resistance	13	75

**FIGURE 8:**
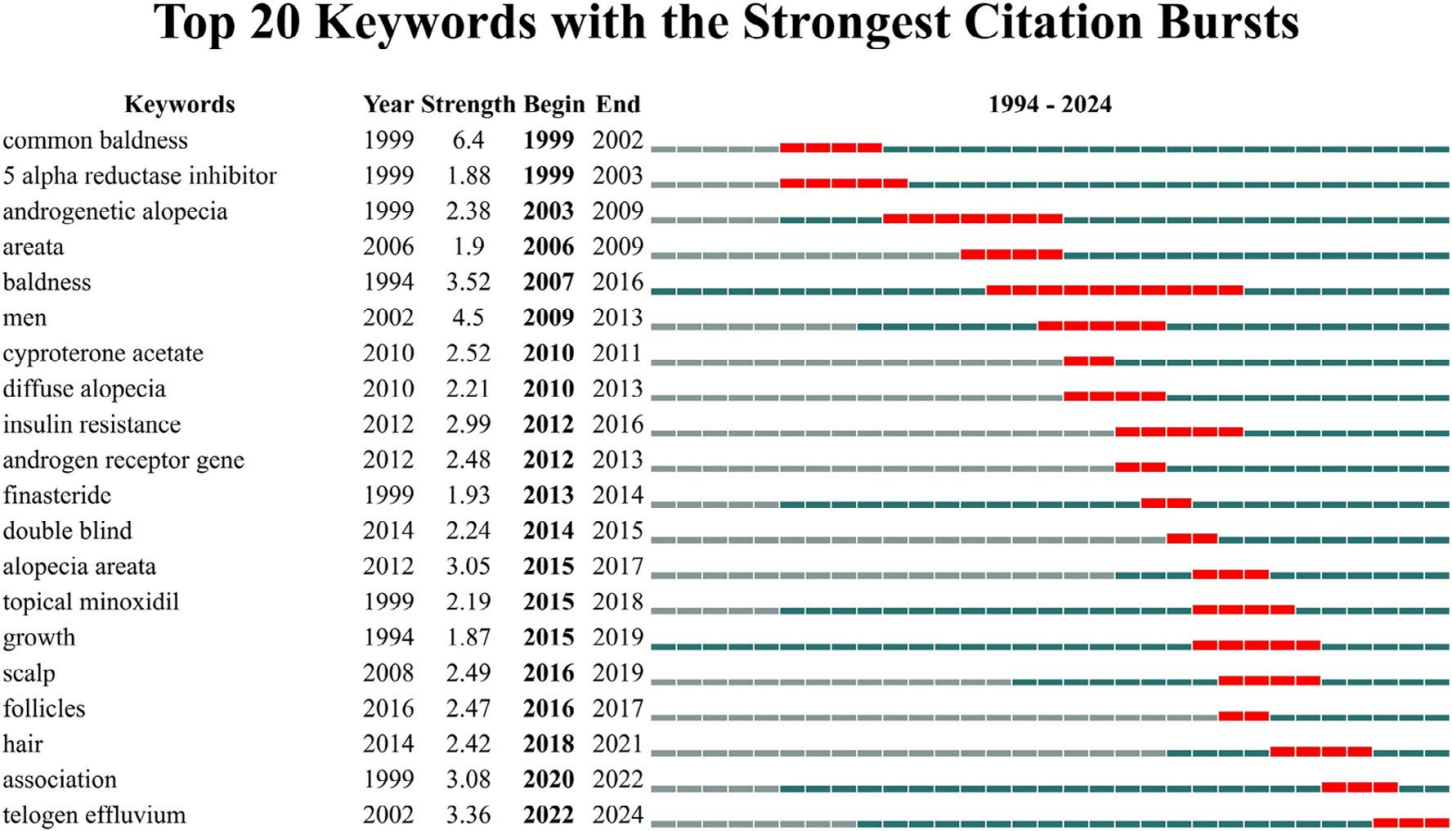
Citation burst analysis of keywords in FPHL.

The analysis of citation bursts, as shown in Figure 8, highlighted the top 20 keywords with the strongest citation bursts, revealing emerging trends within the field. The keyword “common baldness” showed the highest burst strength, starting in 1999 and continuing until 2002, reflecting an early peak in research interest around male‐pattern hair loss. Subsequent bursts included “androgenetic alopecia” from 2003 to 2009 and “baldness” from 2007 to 2016, underscoring a sustained focus on hair loss and related conditions over the years. More recent citation bursts were observed in terms such as “scalp” (2016–2019), “association” (2020–2022), and “telogen effluvium” (2022–2024), indicating a shift toward broader and more varied research topics, including different types of alopecia and hair conditions. These trends suggested a dynamic evolution in research focus, moving from a fundamental understanding of hair loss to more nuanced studies on specific conditions and associated factors (Figure 8).

## 4 Discussion

The analysis revealed notable authorship and global contributions, with 2165 authors publishing 488 documents on this topic. The USA, China, and South Korea emerged as the leading contributors, emphasizing their active role in this research domain. Prominent institutions, including Egyptian Knowledge Bank, University of Melbourne, and National Taiwan University, had contributed substantially, establishing these institutions as key players in advancing the understanding of FPHL. From the perspective of total publications, the *Journal of Cosmetic Dermatology*, *Journal of the American Academy of Dermatology*, and *British Journal of Dermatology* stood out as significant journals in FPHL research, respectively, with papers of great importance and reference value.

### 4.1 Research Hotspots

Keyword, analysis highlights “mechanisms,” “management,” and “safety” as emerging research hotspots in FPHL. However, the mechanisms underlying FPHL remain incompletely understood. Current evidence suggests a multifactorial etiology involving genetics, sex steroid hormones, and environmental factors. FPHL typically manifests in genetically predisposed individuals, characterized by altered hair follicle cycling and follicular miniaturization. This process leads to the transformation of terminal hair follicles into shorter, finer vellus hair follicles, primarily under the influence of androgens [6]. Histologically, it is thought to be caused by a reduction in dermal papilla volume [25]. Consistent with this pathophysiological mechanism, higher levels of dihydrotestosterone and 5‐alpha‐reductase type II are common in FPHL. Although levels of testosterone are similar in individuals with and without FPHL, levels of unbound testosterone, or active testosterone, are higher in those with FPHL [8]. To understanding the pathophysiology of FPHL for precise treatment, more researches should be conducted.

A growing interest was observed in developing and evaluating management options for FPHL, particularly those that address hormonal and metabolic pathways [9, 26]. This finding highlighted the ongoing demand for effective, evidence‐based management strategies for FPHL, which is a common condition that significantly impacts quality of life [27]. Despite its prevalence, FPHL remains challenging to treat due to the shortage of high‐evidence therapeutic options. Topical minoxidil, the first‐line treatment since the 1990s, is currently the only therapy with strong clinical evidence, yet approximately 40% of patients do not respond to it. Anne et al. demonstrated that 5% topical minoxidil was superior to placebo on each of the three primary efficacy end points: promoting hair growth as measured by change in nonvellus hair count and patient/investigator assessments of hair growth and scalp coverage [28]. Besides, low‐dose oral minoxidil has been reported as effective for FPHL [29]. Due to the key role of androgen in FPHL, Finasteride has also been increasingly used as an off‐label treatment for FPHL [30]. Nevertheless, studies on the efficacy of topical finasteride in females have been limited [31], underscoring the need for more multicenter randomized, controlled studies with long‐term follow‐up to evaluate current treatments and explore new therapeutic combinations. For cases unresponsive to conventional therapies, additional clinical, surgical, and camouflage techniques are available, offering alternative approaches to managing this condition [31, 32, 33].

In addition to efficacy, safety of medicine strategies should be considered. The most common adverse effects of topical minoxidil are hypertrichosis in the facial region and local reactions on the scalp, such as pruritus, burning sensation, erythema, papules, or pustules. The frequency of these manifestations depends on the concentration of minoxidil and the type of vehicle, ranging from 1.9% to 5.7% in different studies [34]. Finasteride is generally well‐tolerated. Infrequent side effects in female patients include breast tenderness and increased libido, which are most common in the first year of therapy and tend to diminish with continued use. However, whenever used in premenopausal women, it should always be combined with oral contraceptive pills or another effective method of contraception to prevent inadvertent conception and feminization of the male fetus [25]. Thus, more high‐quality trials should be conducted to evaluate the safety and efficacy of various drugs.

### 4.2 Research Frontiers

The citation burst analysis further emphasizes emerging trends since 2016. The term “scalp” gained prominence in research from 2016 to 2019 as studies increasingly focused on the scalp's microenvironment and its critical role in hair loss mechanisms. Scalp health has emerged as a key factor influencing hair follicle function, directly impacting conditions like FPHL [35]. FPHL is characterized by progressive thinning in a distinct distribution pattern on the scalp, usually without other dermatologic issues. Early recognition and intervention are essential, as timely treatment—both pharmacologic and nonpharmacologic—can slow FPHL's progression and help preserve existing hair. This growing focus on the scalp underscores the importance of targeted therapeutic strategies aimed at optimizing scalp health to manage and potentially mitigate FPHL more effectively [36].

The term “association” gained prominence from 2020 to 2022, highlighting a surge in research examining the links between FPHL and various systemic conditions, such as metabolic syndrome, hormone levels, and genetic factors [37, 38]. This increased interest reflects a growing recognition of the comorbidities and risk factors associated with FPHL, contributing to a more comprehensive understanding of the condition. In this context, a recent study explored the relationship between male androgenetic alopecia (MAGA) and FPHL, finding that a family history, particularly on the maternal side, significantly elevates the risk of developing FPHL alongside MAGA [39]. Among 469 male patients with MAGA, 65.9% also exhibited signs of FPHL. Notably, those with a maternal history of androgenetic alopecia were at a higher risk, underscoring the importance of genetic factors in the manifestation of hair loss across genders. These findings suggest that early screening for FPHL in patients with MAGA and a maternal family history may aid in timely intervention and management, further supporting a holistic approach to understanding and treating hair loss conditions [37].

The recent surge in research on “telogen effluvium” (2022–2024) underscores a growing interest in differentiating this type of hair loss from FPHL, as both share certain clinical features [40, 41]. This attention highlights ongoing efforts to refine diagnostic criteria and gain a clearer understanding of the distinct and overlapping mechanisms underlying different types of hair loss. Vitamin D has emerged as an important factor in hair health, attributed to its anti‐inflammatory and immunomodulatory properties, along with its role in keratinocyte differentiation within hair follicles. Studies have shown an inverse relationship between serum vitamin D levels and non‐scarring hair loss types, including both FPHL and telogen effluvium, suggesting that vitamin D deficiency may contribute to these conditions. Although low vitamin D levels have been associated with various forms of alopecia, including scarring types, additional research is needed to confirm the effectiveness of vitamin D supplementation as a standard treatment for hair loss [42].

### 4.3 Significance and Limitations

This study provides valuable insights into the research landscape of FPHL, offering researchers a guide to identifying current trends, prominent journals, influential authors, and potential collaborators. By mapping research hotspots and recent trends, this bibliometric analysis aids researchers in identifying key areas of focus, understanding the evolution of research topics, and finding suitable platforms for disseminating their work. However, the study has certain limitations. First, it is based on data from a single database, which may exclude relevant studies indexed in other databases. Second, our analysis did not include book chapters, editorials, or conference proceedings, which could provide additional perspectives on the topic. Additionally, the keyword analysis may overlook some terms or concepts that are less commonly used but still relevant, leading to potential gaps in capturing the full breadth of research in this domain.

This study utilized bibliometric and co‐occurrence analyses to evaluate research trends and hotspots within the domain of FPHL. The analysis revealed that research hotspots center on the efficacy and safety of various management strategies against FPHL and its mechanism, and frontier on accurate diagnosis and risk factors related to FPHL. The findings have potential clinical implications by identifying research areas that may translate into improved patients' diagnosis and personalized management.

## Author Contributions

Hongjuan Fu carried out the studies, participated in collecting data, and drafted the manuscript. Hongjuan Fu performed the statistical analysis and participated in its design. Hongjuan Fu helped to draft the manuscript, read and approved the final manuscript.

## Ethics Statement

The author has nothing to report.

## Conflicts of Interest

The author declares no conflicts of interest.

## Data Availability

All data generated or analyzed during this study are included in this published article.
